# Diagnosis of Bacterial Bloodstream Infections: A 16S Metagenomics Approach

**DOI:** 10.1371/journal.pntd.0004470

**Published:** 2016-02-29

**Authors:** Saskia Decuypere, Conor J. Meehan, Sandra Van Puyvelde, Tessa De Block, Jessica Maltha, Lompo Palpouguini, Marc Tahita, Halidou Tinto, Jan Jacobs, Stijn Deborggraeve

**Affiliations:** 1 Telethon Kids Institute, University of Western Australia, Perth, Australia; 2 Biomedical Sciences Department, Institute of Tropical Medicine Antwerp, Antwerpen, Belgium; 3 Clinical Sciences Department, Institute of Tropical Medicine Antwerp, Antwerpen, Belgium; 4 Center for Molecular and Vascular Biology, KU Leuven, Leuven, Belgium; 5 Clinical Research Unit of Nanoro (CRUN), Nanoro, Burkina Faso; 6 Department of Immunology and Microbiology, KU Leuven, Leuven, Belgium; University of Tennessee, UNITED STATES

## Abstract

**Background:**

Bacterial bloodstream infection (bBSI) is one of the leading causes of death in critically ill patients and accurate diagnosis is therefore crucial. We here report a 16S metagenomics approach for diagnosing and understanding bBSI.

**Methodology/Principal Findings:**

The proof-of-concept was delivered in 75 children (median age 15 months) with severe febrile illness in Burkina Faso. Standard blood culture and malaria testing were conducted at the time of hospital admission. 16S metagenomics testing was done retrospectively and in duplicate on the blood of all patients. Total DNA was extracted from the blood and the V3–V4 regions of the bacterial 16S rRNA genes were amplified by PCR and deep sequenced on an Illumina MiSeq sequencer. Paired reads were curated, taxonomically labeled, and filtered. Blood culture diagnosed bBSI in 12 patients, but this number increased to 22 patients when combining blood culture and 16S metagenomics results. In addition to superior sensitivity compared to standard blood culture, 16S metagenomics revealed important novel insights into the nature of bBSI. Patients with acute malaria or recovering from malaria had a 7-fold higher risk of presenting polymicrobial bloodstream infections compared to patients with no recent malaria diagnosis (*p*-value = 0.046). Malaria is known to affect epithelial gut function and may thus facilitate bacterial translocation from the intestinal lumen to the blood. Importantly, patients with such polymicrobial blood infections showed a 9-fold higher risk factor for not surviving their febrile illness (*p*-value = 0.030).

**Conclusions/Significance:**

Our data demonstrate that 16S metagenomics is a powerful approach for the diagnosis and understanding of bBSI. This proof-of-concept study also showed that appropriate control samples are crucial to detect background signals due to environmental contamination.

## Author Summary

Bacterial bloodstream infection (bBSI) is one of the biggest causes of mortality in critically ill patients and standard diagnosis is still done by blood culture methods. Parallel deep sequencing of the 16S ribosomal RNA genes (16S metagenomics) is a new and rapidly evolving research field for profiling bacterial communities. We designed a 16S metagenomics approach for the identification of bacteria in the blood of patients with a bBSI, and evaluated its performance in 75 children with severe febrile illness in Burkina Faso. In addition to superior sensitivity compared to standard blood culture, 16S metagenomics revealed important novel insights into the nature of bBSI. Patients with acute malaria or recently recovered from acute malaria are at increased risk of presenting polymicrobial bloodstream infection, which was in itself a significant risk for non-survival. This proof-of-concept study shows that 16S metagenomics is a powerful approach to diagnose and understand bBSI but also that appropriate control samples are crucial for correct data interpretation.

## Introduction

Bacterial bloodstream infection (bBSI) is one of the biggest causes of mortality in critically ill patients [1,2]. Rapid and accurate diagnosis of bBSI is crucial for the survival of the patient. The reference diagnostic test for bBSI is *in vitro* culture of the blood followed by identification of the cultured bacteria. However, blood culture shows a maximum sensitivity of 60% [3], is labor intensive and takes several days before the results are available. Furthermore, empirical treatment with antibiotics prior to hospital admission jeopardizes bacterial growth in culture, further decreasing the sensitivity of the test.

Molecular techniques have evolved in the last decades as an alternative for blood culture in the diagnosis of bBSI. Most molecular diagnostics are based on the detection of bacterial DNA in the blood using polymerase chain reactions (PCR) and require different PCR tests for every bacterial species [4]. Several multiplexed PCR formats for the parallel detection of different bacteria in blood samples have been developed [5–7]. PCR testing is faster than blood culture but the sensitivity is often in the same range as blood culture and shows low reproducibility. There is also a limit to the number of bacterial species that can be simultaneously tested, since multiplexing of different PCR assays is technically challenging due to differences in amplification efficiencies of the different primer sets and the limited number of available fluorescent labels. In contrast to PCR, 16S metagenomics provides a so-called assumption-free detection method, meaning that the method is not targeting one specific bacterial species but can detect all the different bacteria present in a clinical sample in one single assay. 16S metagenomics is based on parallel deep sequencing of the prokaryote-specific 16S ribosomal RNA gene [8,9]. This gene has conserved and variable sequence regions. The conserved sequence allows pre-amplification of the variable region using a single primer set, where after the species can be identified based on the sequence read of the variable region. 16S metagenomics is frequently used to profile bacterial communities in the environment and the human gut [9,10]. While Frickmann *et al*. applied 16S metagenomics on the supernatant of blood cultures [11], two recent studies used 16S metagenomics directly on blood. Faria *et al*. developed a 16S metagenomics method for whole blood and applied their method to three septic patients from an intensive care unit [12]. Gyarmati *et al*. applied 16S metagenomics on the blood of 33 neutropenic patients at a European University Hospital and reported a high diversity of bacteria [13].

In this study, we applied 16S metagenomics to identify bacteria in the blood of 75 children with severe febrile illness in Nanoro, Burkina Faso. We present the design of the 16S metagenomics assay, the diagnostic performance compared to blood culture, and the potential impact on the diagnosis and study of bloodstream infections.

## Methods

### Ethics statement

Ethical approval was obtained from the ethics committees of the Institute of Tropical Medicine Antwerp and the University Hospital Antwerp in Belgium and the national ethics committee of Burkina Faso. Written informed consent was given by the parents or guardians of all children.

### Patients

Seventy-five children (median age 15 months), admitted to hospital or health centre, with severe febrile illness were recruited at the Saint Camille District Hospital or CSPS Urbain in Nanoro, Burkina Faso, between January and April 2013 [14]. Severe febrile illness was defined as an axillary temperature of at least 38°C, and/or clinical signs associated with bloodstream infection such as respiratory distress, prostration, altered consciousness, convulsions, clinical jaundice, hypothermia, signs of shock, severe malnutrition (weight for height score < 70% according to national guidelines or kwashiorkor) or with severe anemia (hemoglobin < 5 g/dl). Venous blood for blood culture was collected. In case of suspicion of bacterial meningitis, lumbar punctures were performed. Patients were treated based on *in vitro* culture results and according to national guidelines with ampicillin and gentamicin for neonatal sepsis and ceftriaxone for sepsis or meningitis in older children. Malaria was diagnosed by blood films stained with 3% Giemsa solution (pH 7.2) and the malaria SD Bioline Pf rapid diagnostic test (Standard Diagnostics, Hagal-Dong, Korea).

### Blood culture

bBSI were diagnosed on site by collecting 1–3 ml of venous blood into pediatric blood culture bottles (BD BACTEC Peds PlusTM/F, Becton Dickinson and Company, Maryland, USA), which were incubated for 5 days in a BACTEC 9050 incubator (Becton Dickinson). Positive blood cultures were Gram stained, sub-cultured on Eosin-Methylene blue (EMB) agar and 5% sheep blood agar, and incubated at 35–37°C for 24 hours in respectively atmospheric and 5% CO_2_ environments. Bacterial isolates were identified to the species level by standard biochemical methods. EDTA blood samples were used for the diagnosis of malaria and the left-over blood was stored at -80°C for maximum one year and shipped frozen to the Institute of Tropical Medicine Antwerp (Belgium) for metagenomics analysis. Quality indicators used for monitoring of blood culture performance included blood volume sampled, proportion of contaminants and proportions of clinically significant bacteria recovered. In addition, pathogens were confirmed at the Institute of Tropical Medicine Antwerp for identification and antibiotic susceptibility testing [14].

### 16S metagenomics

Blood sample volumes available for 16S metagenomics varied between 200 and 1000 μl, with an average of 524 μl. Blood was mixed with equal volumes of lysozyme solution, containing 40 mg lysozyme (Sigma Aldrich) in 20 mM Tris-HCl, 2 mM EDTA and 1.2% Triton X (pH 6.2) and incubated at 37°C for 1 hour at 250 rpm. DNA was extracted using the Qiagen Blood Midi Kit (Qiagen, Hilden, Germany) following the manufacturer’s instructions and eluted in 100 μl elution buffer provided with the kit. The V3–V4 region of the bacterial 16S rRNA gene was amplified with the universal primers reported by Klindworth *et al*. [15], fused with Illumina adapter overhang nucleotide sequences. Primer sequences were 5’- TCGTCGGCAGCGTCAGATGTGTATAAGAGACAGCCTACGGGNGGCWGCAG-3’ and 5’ GTCTCGTGGGCTCGGAGATGTGTATAAGAGACAGGACTACHVGGGTATCTAATCC-3’. Three independent PCR reactions were performed for each sample. The products were pooled and indexed using Illumina’s 16S Metagenomic Sequencing Library Preparation protocol (Illumina, San Diego CA). Libraries were deep sequenced with the Illumina MiSeq sequencer using a reagent v3 cartridge and 600 cycles. Four negative control blood samples from a healthy Belgian volunteer were taken along the complete procedure. Using MOTHUR v1.34.1 [16], paired reads were combined into single contigs. Short (<50 bp), long (>600 bp) and reads with ambiguous nucleotide calls were removed. Reads were taxonomically labeled by a rank-flexible method with GAST [17] and the associated full length 16S SILVA reference database. Reads only called up to genus level were removed, except for *Prevotella* and *Klebsiella*, which are genera predominantly comprising clinically relevant species. Sequence reads corresponding to bacterial species detected in at least one negative control sample were considered to originate from environmental contaminants and thus removed from the analysis; with the following exceptions: *Salmonella enterica*, *Escherichia coli*, *Enterobacter cloacae* and *Shigella dysenteriae*, since they are known pathogens. The 16S metagenomics assay was repeated starting from the DNA extracts and 16S metagenomics species identifications were only considered reproducible and valid when replicated in both runs. One *E*. *coli* case was considered as a positive case based on one single run because of the high abundance of assigned reads (>20000). Samples with positive blood culture and/or positive results in the duplicated 16S metagenomics assay were classified as confirmed bBSI and possible bBSI. Confirmed bBSI was defined as patients in whom bacteria were detected known to cause bBSI. Possible bBSI was defined as patients in whom bacteria were detected that rarely cause bBSI. Classification of patients into confirmed and possible bBSI was done by two independent readers, a clinical microbiologist and an infectious diseases physician.

### Statistical analysis

Pearson’s chi-square test, odds ratio, 95% confidence intervals and corresponding chi-square Mantel-Haenszel *p*-values were calculated in Epi Info 71.5.2 (freeware from CDC, Atlanta, USA).

## Results

### Blood culture versus 16S metagenomics

**Fig 1:**
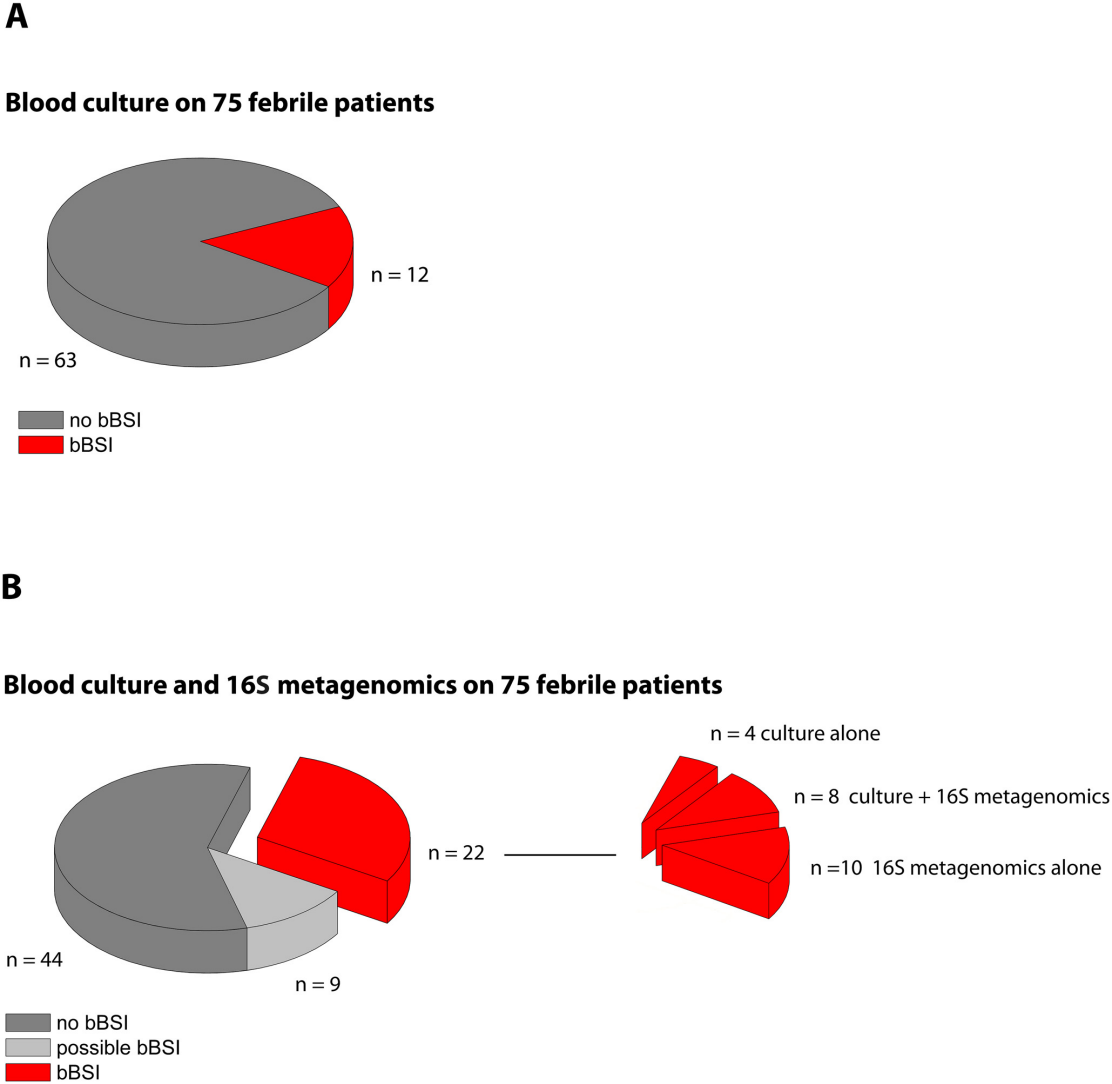
Numbers of patients classified as confirmed bacterial bloodstream infection (bBSI) based on (A) blood culture alone and (B) combined data from blood culture and 16S metagenomics.

Blood cultures grew clinically significant bacteria for 12 out of the 75 patients (16%) (Fig 1A). In comparison, 16S metagenomics detected clinically significant bacteria in 18 patients (24%), including 8 of the 12 blood culture positive patients and 10 patients with a negative blood culture. In total, 22 patients (29.3%) were classified as confirmed bBSI by blood culture and/or 16S metagenomics (Fig 1B). In addition, 9 patients were classified as possible bBSI because potentially clinically relevant bacteria were identified by the replicated 16S metagenomics assay. For 51 samples, the repeated 16S assay generated reproducible reads that could only be identified to genus level and no reproducible reads that could be identified to species level. Since we did not consider reads called up to genus level only, all these 51 samples were considered negative for bBSI. The bacteria identified at genus level were not clinically relevant (*e*.*g*. *Acinetobacter*, *Aerococcus*, *Shewanella*, *Rhodanobacter*, *Pusillomonas*, *Flacklamia*, *Petrobacter*, *Geobacillus*, *Nocardioides*, *Filimonas*, *Propionispira*, *Lysobacter*, *Tissierella*, *Leptotrichia*, *Paludimonas*, *Kineosphaera*, *Raoultella*, *Proteiniphilum*, *Amaricoccus*, *Luteimonas*). The bacterial species identified in the negative control samples during the 2 analyses are detailed in S1 File. In the first round of sequencing we detected 1 read of Salmonella enterica in 2 different negative control samples and 1 read of *Shigella dysenteriae*, *Escherichia coli* and *Enterobacter cloacae* in 3 different negative control samples. In the second round of sequencing on the same DNA extracts, we detected 1 read of *Escherichia coli* and *Shigella dysenteriae* in 1 negative control sample. In contrast to the patient samples, the detection of these pathogenic species in the negative control samples was not reproducible across the two experiments. In addition, these species were each time only detected as a single read (S1 Table) further indicating that the appearance of those bacterial species in the negative control samples is most likely due to spurious taxonomically labelling. For 29 samples, we detected clinically relevant bacterial species in only one of the 2 repeated runs. Since the results could not be reproduced in the second run, they were classified as negative for bBSI.

### Bacterial species causing bBSI in the patient population

An overview of the bacteria identified in the 22 patients classified as confirmed bBSI is presented in Table 1. The detailed results of the replicated 16S metagenomics assays are presented in S1 Table. *Salmonella enterica* was detected in half of the confirmed bBSI patients, while *Escherichia coli* was detected in 4 cases and *Shigella* spp., *Neisseria meningitidis* and *Streptococcus pneumoniae* in 3 cases. Less commonly reported bacteria such as *Morganella morganii*, *Aeromonas media*, and *Pantoea agglomerans* were detected in combination with known clinically significant bacteria. Although the sample size used in this study was small, our results further suggest that the 16S metagenomics assay had a significant better diagnostic performance than blood culture in antibiotic pre-treated patients. Out of the 27 patients that reported antibiotics use prior to study enrollment, 4 tested positive by blood culture compared to 11 with the 16S metagenomics assay (Pearson’s Χ^2^ = 4.52, p-value = 0.03) The 16S metagenomics assay detected multiple Gram-negative bacterial species in 9 out of the 22 confirmed bBSI cases, including 6 cases with 2 species, 2 cases with 3 species and 1 case with 4 different species. Interestingly, we observed that the odds are almost 7-fold higher for confirmed bBSI patients with acute malaria or recently recovered from acute malaria to carry multiple Gram-negative species in the blood, compared to bBSI patients with no recent malaria history (Table 2). In the 9 patients classified as possible bBSI, the 16S metagenomics assay detected *Enterobacter aerogenes* in 6 patients, of which 1 patient was also positive for *Corynebacterium mycetoides* and a second patient also carried DNA of *Corynebacterium segmentosum* and *Prevotella spp* (Table 3).

**Table 1:** Bacterial diversity in the blood of the 22 patients classified as confirmed bBSI based on the results of blood culture and 16S metagenomics.

Patient	Malaria	Prior antibiotics	Survival	Blood culture	16S Metagenomics			
					Bacterium 1	Bacterium 2	Bacterium 3	Bacterium 4
1	yes	no	yes		*Salmonella enterica*			
2	yes	no	yes		*Salmonella enterica*			
3	no	no	yes		*Salmonella enterica*			
4	yes	no	yes		*Salmonella enterica*			
5	no	no	yes	*Salmonella enterica*				
6	no	no	yes	*Staphylococcus aureus*	*Staphylococcus aureus*			
7	no	no	yes	*Streptococcus pneumoniae*				
8	no	Ceftriaxone	yes		*Streptococcus pneumoniae*			
9	no	no	yes		*Bordetella pertussis*			
10	no	no	yes	*Neisseria meningitidis*				
11	no	no	no	*Neisseria meningitidis*				
12	no	Ampicillin	yes		Neisseria meningitides*			
13	no	Ampicillin	no		Streptococcus pneumoniae*			
14	yes	no	yes	*Escherichia coli*	*Escherichia coli*	*Salmonella enterica*		
15	no	no	no	*Salmonella enterica*	*Salmonella enterica*	*Enterobacter cloacae*		
16	no	no	unknown	*Haemophilus influenzae*	*Haemophilus influenzae*	*Salmonella enterica*		
17	yes	no	yes		*Salmonella enterica*	*Morganella morganii*		
18	yes	Ampicillin	no	*Salmonella enterica*	*Salmonella enterica*	*Aeromonas media*		
19	yes	no	yes		*Escherichia coli*	*Shigella* spp.		
20	yes	Amoxicillin	no	*Escherichia coli*	*Escherichia coli*	*Shigella* spp.*	*Enterobacter aerogenes*	
21	no	Cotrimoxazole	no	*Escherichia coli*	*Escherichia coli*	*Shigella* spp.*	*Rickettsia bellii*	
22	no	Cotrimoxazole	no	*Salmonella enterica*	*Salmonella enterica*	*Enterobacter cloacae*	*Klebsiella* spp.	*Pantoea agglomerans*

* comprises multiple closely related species as detailed in S1 Table.

**Table 2:** Association multiple species bBSI, malaria history and mortality in confirmed bBSI patients.

EXPOSURE	cases	controls	OUTCOME	cases	controls	Odds ratio	95% CI
malaria history	9	13	multiple species	9	13	6.67	1.00–44.29
multiple species*	8	13	mortality*	7	14	9.17	1.15–73.24
malaria history*	9	12	mortality*	7	14	1.00	0.16–6.36

* For 1 patient left against medical advice, no disease outcome information available.

**Table 3:** Bacterial diversity in the blood of the 9 patients classified as possible bBSI patients based on 16S metagenomics.

Patient	Malaria	Prior antibiotics	Survival	16S metagenomics	
				Bacterium 1	Bacterium 2
1	yes	Ampicillin	Yes	*Prevotella* spp.	
2	yes	no	Yes	*Aeromonas media*	
3	yes	no	Yes	*Yersinia enterocolitica*	
4	yes	Ampicillin	Unknown	*Enterobacter aerogenes*	
5	no	Cotrimoxazole	Yes	*Enterobacter aerogenes*	
6	yes	no	Yes	*Enterobacter aerogenes*	
7	yes	no	Yes	*Enterobacter aerogenes*	
8	yes	no	Yes	*Enterobacter aerogenes*	*Corynebacterium mycetoides*
9	no	Ampicillin	yes	*Enterobacter aerogenes*	*Corynebacterium segmentosum*

### bBSI diagnosis and mortality

Seven out of the 22 confirmed bBSI patients did not survive their illness (46.7%), while this was only 20% for the complete study group. Interestingly, the 10 confirmed bBSI cases that were missed by blood culture included only 1 non-survival patient, and culturing of the cerebrospinal fluid of this patient revealed bacterial meningitis caused by *Streptococcus pneumoniae*. These findings suggest that blood culture is sensitive enough to pick up the most severe cases, and the additional cases identified by the 16S metagenomics assay are predominantly cases that subsequently have a good clinical outcome. Importantly, in 5 out of the 7 non-survival bBSI patients more than 1 Gram-negative bacterial species was detected. Our results suggest that the odds for bBSI patients with multiple Gram-negative bacterial species in the blood to have poor clinical outcome are 9-fold higher than in bBSI patients in which just one bacterial species is detected (Table 2).

## Discussion

We report the outcome of a proof-of-concept study that uses 16S metagenomics on whole blood for the identification of bacteria in blood of severely ill febrile patients. A 16S metagenomics workflow was developed for bBSI diagnosis and compared with conventional blood culture for 75 children with severe febrile illness in Nanoro, Burkina Faso. Out of the 75 patients included in our study, clinically significant bacteria were detected in 12 patients by blood culture of which 8 were also detected by 16S metagenomics, delivering the proof-of-concept of our approach. The 4 blood culture positive patients who were negative in 16S metagenomics were presumably missed due to the low volumes of blood available for DNA extraction. The volume of blood for bBSI diagnosis is particularly critical since the bacterial load in the blood during bBSI can be as low as 1 colony-forming unit per ml of blood [18]. 16S metagenomics identified pathogenic bacteria in 10 additional patients that were negative by blood culture, thus almost doubling the bBSI positive fraction in our study cohort. The 16S metagenomics assay diagnosed more bBSI than blood culture in patients who reported to have used antibiotics prior to blood collection. Residual antibiotic molecules in the blood may inhibit bacterial growth during blood culture, while 16S metagenomics is entirely based on bacterial DNA detection. We conclude that the combination of blood culture and 16S metagenomics has a superior sensitivity to identify bBSI patients compared to blood culture alone.

The 16S metagenomics assay allowed us to detect more than one bacterial species in almost half of the confirmed bBSI patients, a finding which was completely missed by blood culture. We observed that bBSI patients with acute malaria or recovering from malaria have a significant 7-fold higher risk to carry multiple Gram-negative bacteria in their blood compared to bBSI patients with no recent malaria history. The 7 confirmed bBSI patients with bacteria known to cause a specific pathology on their own (4 Gram-positive bBSI, 3 *Neisseria* spp. bBSI), did not have a recent malaria history and did not carry additional bacterial species. Our data suggest a causal pathway whereby malaria history and multiple bacteremia contribute to bBSI mortality. A malaria history puts the patient at a higher risk to develop bBSI with multiple species (OR = 6.67), which in turn is associated with higher mortality (OR = 9.17). In contrast, malaria history alone in a bBSI patient does not put that patient at higher risk for bBSI mortality (OR = 1.0). It must be emphasised that the available sample size is too small to draw any definite conclusions on how the different factors relate to each other and larger studies are needed to further investigate the robustness of this relationship. Bacterial bloodstream infection is increasingly recognized as a complication in children with severe *Plasmodium falciparum* malaria. Church and Maitland recently conducted a systematic review of 25 studies across 11 African countries and reported that children with recent or acute malaria are at an increased risk of bBSI, which results in an increased risk of mortality [19]. Although the majority of bacterial pathogens involved were enteric Gram-negative organisms in particular non-typhoidal *Salmonella* (NTS), the exact nature of the interaction between malaria and bBSI is still not clear. Wilairatana et al. reported that *P*. *falciparum* patients show increased gut permeability [20], which may facilitate bacterial translocation from the intestinal lumen to the systemic circulation. Gómez-Pérez postulated that malaria can lead to a dysfunctional spleen in young children increasing the risk to develop bBSI [21]. Other hypotheses are macrophage dysfunctions due to reduced cytokine production after ingestion the malaria pigment haemozoin [22], competition between bacteria and damaged red blood cells for phagocytic sites [21], and reduced production of cytokines such as IL-12 [23]. Cunnington et al. observed that *P*. *falciparum* infection in mice impairs the neutrophil oxidative burst and thus resistance to *Salmonella* infection [24]. The detection of translocated bacteria from the gut into the blood has clinical significance as it indicates critical illness. Indeed, we observed that confirmed bBSI patients with multiple Gram-negative bacterial species in the blood showed a significant 9-fold higher risk for poor clinical outcome compared to patients in which only a single bacterial species was detected. Seven out of the 9 patients classified as possible bBSI were also acute or recent malaria patients. Hence it is possible that the detected bacteria in these patients, which were unusual bBSI agents for our study settings, may also originate from the gut. However we should interpret the results of the possible bBSI patients with care as we cannot exclude that some of the detected bacteria correspond to environmental contaminants picked up during sample collection and work-up.

The power of 16S metagenomics is that it provides an assumption-free approach to identify bacteria. However, the broad range of bacteria that can be detected limits the specificity of the assay. First of all, it can be unclear whether the detected bacteria are truly clinically significant or merely environmental contaminants picked up during sample preparation, as was the case with the possible bBSI patients. We expect that including quality control samples that are collected at the same time and location as the index samples will largely resolve this issue. Secondly, species such as *E*. *coli* and different *Shigella* spp. have highly similar 16S rRNA genes and the separation of such closely related species based upon a subsection of this gene can be less reliable. This is further complicated by the fact that several bacteria show intra-genomic heterogeneity in the sequences of their 16S rRNA genes [25]. Confirmatory genotyping tests based on additional genomic markers may thus be advisable in situations where high specificity is required.

Some limitations in our study should be taken into account. The negative control samples used to filter out environmental contaminants were collected from a different location and at a different time point than the patient samples. In addition, the blood samples tested were left-over samples from tubes that had been accessed for other diagnostic tests, potentially introducing environmental contaminants. The lack of appropriate controls made it difficult to distinguish such environmental contaminants from clinically relevant bacteria. For future 16S metagenomics studies on blood samples, we suggest that blood samples should be collected in dedicated tubes for 16S metagenomics and that control samples are collected at the same place and time as the patient samples. Secondly, the blood sample volumes that were available for 16S metagenomics may have been too low for some patients to allow accurate detection of bacteria. Thirdly, no data of other molecular tests, such as diagnostic PCRs, are available for our study samples. Fourthly, read depths of pathogens varied largely between the replicate 16S metagenomics runs and this may have affected the reproducibility of our 16S metagenomics assay. This is probably due to the limited blood volumes tested, possible amplification bias by PCR during the library preparation [26] and the inherent variation between deep sequencing runs [27].

In conclusion, our proof-of-concept study shows that 16S metagenomics is a powerful approach to identify bacteria in the blood of patients and complementary to microbiological culture based approaches. It provides an assumption-free method to study invasive bacterial disease and can detect multiple bacterial species present in a blood sample in one single run. Our data show that 16S metagenomics has the potential to revolutionize the diagnosis of bBSI in reference hospitals with molecular biology facilities and surveillance studies mapping bBSI incidences.

## Supporting Information

S1 Checklist STARD checklist. (PDF)

S1 Flowchart STARD flowchart. (PDF)

S1 File GAST output with taxonomic labeling of the 16S metagenomics reads in the 75 blood samples. (ZIP)

S1 Table Detailed results of survival, malaria diagnosis, use of prior antibiotics, blood volume used for DNA extraction, blood culture, and replicated (run 1 and run 2) 16S metagenomics for the 75 patients. (PDF)
