# Correction: Diagnosis of Bacterial Bloodstream Infections: A 16S Metagenomics Approach

**DOI:** 10.1371/journal.pntd.0005811

**Published:** 2017-07-28

**Authors:** Saskia Decuypere, Conor J. Meehan, Sandra Van Puyvelde, Tessa De Block, Jessica Maltha, Lompo Palpouguini, Marc Tahita, Halidou Tinto, Jan Jacobs, Stijn Deborggraeve

There is an error in the first sentence of the “bBSI diagnosis and mortality” subsection within the Results. The correct sentence is: Seven out of the 22 confirmed bBSI patients did not survive their illness (31.8%), while this was only 20% for the complete study group (15 out of 75).
