# Supplementary figures and images for: Diagnosis of Bacterial Bloodstream Infections: A 16S Metagenomics Approach
Source: PLoS Negl Trop Dis. 2016 Feb 29;10(2):e0004470. doi: 10.1371/journal.pntd.0004470 (PMC4771206; doi:10.1371/journal.pntd.0004470)

General example

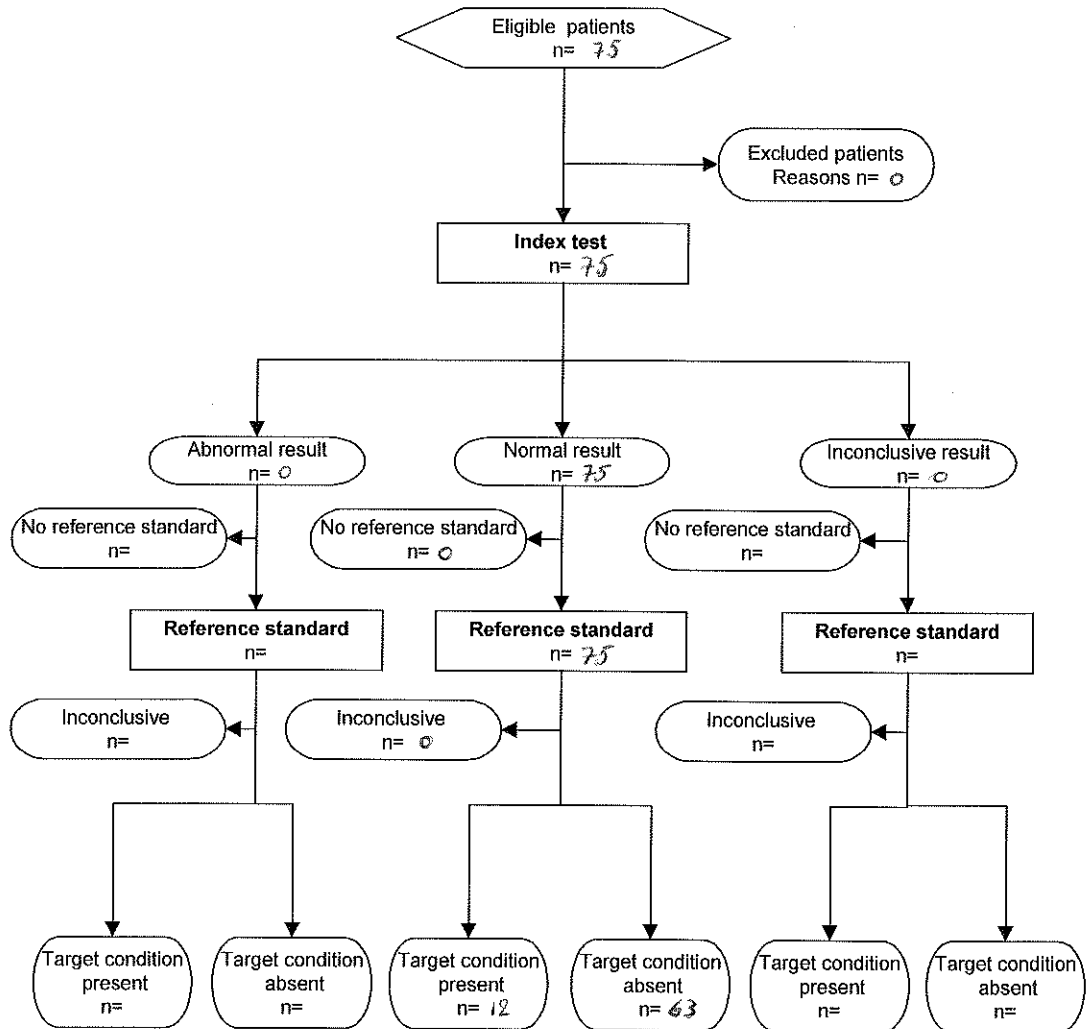

Supplement: S1 Flowchart — (PDF) [file pntd.0004470.s002.pdf]
