# Supplementary material for: Diagnosis of Bacterial Bloodstream Infections: A 16S Metagenomics Approach
Source: PLoS Negl Trop Dis. 2016 Feb 29;10(2):e0004470. doi: 10.1371/journal.pntd.0004470 (PMC4771206; doi:10.1371/journal.pntd.0004470)
Supplement: S1 Table — (PDF) [file pntd.0004470.s004.pdf]

Supplemental table S1. Detailed results of survival, malaria diagnosis, use of prior antibiotics, blood volume used for DNA extraction, blood culture, and replicated (run 1 and run 2) 16S metagenomics for the 75 patients.

								blood culture	16S metagenomics														
patient	survival	malaria	malaria thick blood smear	malaria SD Bioline Pf RDT	prior antibiotics	µl blood extracted	bBSI based on culture and/or two 16S metagenomics runs	bacterium	bacterium 1	reads run1	reads run2	bacterium 2	reads run1	reads run2	bacterium 3	reads run1	reads run2	bacterium 4	reads run1	reads run2		reads run1	reads run2
MCMA429	no	yes	pos	pos	neg	400	no		Acinetobacter parvus	2	1												
MCMA430	yes	yes	pos	pos	pos	600	no																
MCMA431	yes	yes	pos	pos	neg	750	no																
MCMA433	yes	yes	pos	pos	pos	200	possible		Prevotella spp	20	7												
MCMA434	yes	no	neg	neg	pos	200	yes		Streptococcus pseudopneumoniae	123	2												
MCMA435	yes	yes	pos	pos	neg	700	yes		Escherichia coli	21942	0	Shigella flexneri	7977	0									
MCMA436	unknown	no	neg	neg	neg	1000	yes	Haemophilus influenza	Haemophilus influenzae	7	2	Salmonella enterica	2	1									
MCMA437	yes	no	neg	neg	pos	500	no																
MCMA440	yes	no	neg	neg	pos	300	no																
MCMA441	yes	no	neg	neg	neg	400	no																
MCMA442	yes	yes	pos	pos	neg	500	no																
MCMA443	yes	yes	neg	pos	neg	300	possible		Corynebacterium mycetoides	1	1	Enterobacter aerogenes	1	1									
MCMA444	no	yes	neg	pos	neg	700	yes	Salmonella Enteritidis	Salmonella enterica	17869	5716	Enterobacter cloacae	37	148									
MCMA445	yes	no	neg	neg	pos	550	no																
MCMA446	yes	yes	neg	pos	neg	600	yes	Escherichia coli	Salmonella enterica	5	4	Escherichia coli	4	3									
MCMA447	yes	yes	pos	pos	pos	750	no																
MCMA448	yes	yes	pos	pos	neg	600	no																
MCMA449	yes	yes	neg	pos	neg	300	yes		Salmonella enterica	2	1	Morganella morganii	24	4									
MCMA451	yes	yes	pos	pos	neg	450	possible		Aeromonas media	5	1												
MCMA452	yes	yes	pos	pos	neg	750	yes		Salmonella enterica	6	1												
MCMA453	no	no	neg	neg	neg	300	no																
MCMA455	no	no	neg	neg	pos	350	yes	Salmonella enteritidis	Salmonella enterica	77071	35902	Enterobacter cloacae	154	711	Klebsiella spp.	2	2	Pantoea agglomerans	2	9			
MCMA458	yes	no	neg	neg	neg	300	no																
MCMA459	yes	no	neg	neg	pos	750	no																
MCMA460	unknown	yes	neg	pos	pos	200	possible		Enterobacter aerogenes	4	4												
MCMA462	yes	yes	neg	pos	neg	300	no																
MCMA463	no	no	neg	neg	pos	750	no																
MCMA464	yes	yes	neg	pos	pos	350	no																
MCMA465	no	yes	neg	pos	pos	500	yes	Salmonella Typhimurium	Salmonella enterica	102	39	Aeromonas media	3	1									
MCMA466	yes	yes	pos	pos	neg	350	yes		Salmonella enterica	5	1												
MCMA468	yes	no	neg	neg	pos	750	no																
MCMA469	yes	no	neg	neg	pos	600	possible		Enterobacter aerogenes	9	2												
MCMA470	yes	no	neg	neg	pos	800	no																
MCMA472	yes	yes	pos	pos	neg	400	no																
MCMA473	yes	no	neg	neg	pos	450	possible		Corynebacterium segmentosum	2	5	Enterobacter aerogenes	3	6	Prevotella spp	1	7						
MCMA474	yes	yes	pos	pos	pos	650	no																
MCMA475	yes	no	neg	neg	pos	600	no																
MCMA476	yes	no	neg	neg	neg	550	yes		Salmonella enterica	7	3												
MCMA477	yes	yes	pos	pos	neg	1000	no																
MCMA478	yes	yes	neg	pos	pos	1000	no																
MCMA480	yes	no	neg	neg	neg	1000	yes	Staphylococcus aureus	Staphylococcus aureus	2	2												
MCMA484	no	no	neg	neg	neg	400	no																
MCMA489	yes	yes	pos	pos	neg	250	no																
MCMA491	yes	yes	neg	pos	neg	700	no																
MCMA493	yes	yes	pos	pos	neg	350	yes		Salmonella enterica	7	3												
MCMA495	yes	yes	pos	pos	neg	300	no																
MCMA497	yes	yes	pos	pos	neg	650	no																
MCMA501	yes	yes	pos	pos	neg	650	no																
MCMA502	yes	yes	pos	pos	neg	350	no																
MCMA513	yes	yes	pos	pos	neg	500	possible		Yersinia enterocolitica	2	1												
MCMA515	yes	no	neg	neg	neg	700	yes	Salmonella															
MCMA516	yes	yes	pos	pos	neg	450	no																
MCMA520	yes	no	neg	neg	neg	900	yes		Bordetella pertussis	5	6												
MCMA521	yes	yes	neg	pos	pos	250	no																
MCMA523	yes	yes	pos	pos	neg	1000	no																
MCMA526	no	yes	neg	pos	pos	200	yes	Escherichia coli	Escherichia coli	38	18	Shigella flexneri	10	4	Shigella sonnei	1	1	Enterobacter aerogenes	7	1			
MCMA528	no	no	neg	neg	pos	400	yes	Escherichia coli	Escherichia coli	166	115	Shigella flexneri	48	34	Shigella sonnei	23	11	Shigella dysenteriae	5	2	Rickettsia bellii	6	8
MCMA532	yes	no	neg	neg	neg	1000	no																
MCMA533	no	yes	pos	pos	neg	500	no																
MCMA534	yes	yes	neg	pos	pos	800	no																
MCMA535	yes	no	neg	neg	neg	650	yes	Neisseria meningitidis															
MCMA537	no	yes	neg	pos	pos	650	no																
MCMA547	yes	no	neg	neg	pos	500	yes		Neisseria meningitidis	7	4	Neisseria polysaccharea	7	2									
MCMA550	no	yes	pos	pos	neg	400	no																
MCMA557	no	no	neg	neg	neg	350	yes	Neisseria meningitidis															
MCMA560	yes	yes	pos	pos	neg	350	no																
MCMA562	yes	yes	pos	neg	neg	250	no																
MCMA564	no	no	neg	neg	neg	350	no																
MCMA565	yes	no	neg	neg	neg	600	yes	Streptococcus pneumoniae															
MCMA566	no	no	neg	neg	pos	450	yes		Streptococcus pneumoniae	173	11	Streptococcus pseudopneumoniae	90	19									
MURB082	yes	yes	pos	pos	neg	350	possible		Enterobacter aerogenes	4	5												
MURB083	yes	no	neg	neg	neg	250	no																
MURB085	yes	yes	pos	pos	neg	250	possible		Enterobacter aerogenes	9	5												
MURB088	yes	yes	pos	pos	neg	500	no																
MURB095	yes	yes	pos	pos	neg	550	no																
